# Assessment of the structural validity of the domestic violence healthcare providers’ survey questionnaire using a Nigerian sample

**DOI:** 10.5249/jivr.v2i2.41

**Published:** 2010-06

**Authors:** Ime A John, Stephen Lawoko

**Affiliations:** ^*a*^Division of Social Medicine, Department of Public Health Sciences, Karolinska Institutet. SE-17176 Stockholm, Sweden.

## Abstract

**Background:**

There has been increased advocacy to involve healthcare providers in the prevention of intimate partner violence (IPV) through screening for it in healthcare. Yet, only one in ten providers screen for IPV, suggesting barriers. Understanding the readiness of healthcare providers to screen for IPV is therefore paramount. The Domestic Violence Healthcare Provider Survey Scales (DVHPSS) is a previously validated, comprehensive scale to study readiness of healthcare providers to screen for IPV. However, an understanding of its usefulness in the Sub-Saharan African context remains elusive. The current study undertook to examine the structural validity of the DVHPSS in Nigeria.

**Methods:**

Exploratory factor analysis and Cronbach's Alpha were run to reveal the factorial structure and reliability of the instrument/subscales respectively. Established thresholds were used to determine significant factor loadings and alphas coefficient.

**Results:**

A six factor model emerged, with 2 factors similar to the original scale, another two differing slightly and a further two factors resulting from a splitting up of the original combination of victim/provider safety to having distinct victim and provider safety subscales.

**Conclusions:**

With slight modifications, the DVHPSS can be use to study IPV screening among Nigerian healthcare professionals. Introducing screening protocols could promote better understanding of crucial questions that were lost in the analysis.

## Introduction

 Intimate partner violence (IPV) against women remains a public health concern worldwide despite significant law reforms aimed at curbing abuse in various countries.^1-3^ This is indicative of the fact that secondary prevention measures on their own may not be reaching the desired goal. Resources thus should be redirected towards primary prevention. Healthcare professionals can play an important role in this regard through screening for IPV among their patients. Over the past decade, a number of instruments to assist healthcare providers in screening for IPV have been developed, particularly in Europe and America.^4-10^ Despite these developments, barely 10% of health care providers screen for IPV in those settings evidencing barriers to screening for IPV in healthcare.^11,12^ Barriers to screening could evolve from the provider or from the client. An assessment of providers’ readiness to screen for IPV as well as Clients readiness to be screened for IPV thus seems paramount before effective screening can be realized. In this paper, emphasis is laid on the former.

A number of instruments have emerged in the past decade to assess providers’ readiness to screen for IPV.^13,14^ Among the most comprehensive of them is the Domestic Violence Healthcare Provider Survey Scales (DVHPSS).^15^ The scale measures healthcare professionals’ readiness to screen in terms of their perceived knowledge/ efficacy in screening, conflicting professional roles, availability of social support networks to which IPV victims can be referred, whether inquiries into IPV may pose safety challenges for patient/care providers and providers’ general attitudes towards screening for IPV. The DVHPSS has been validated in the western context but to the best of our knowledge, is not yet in use in the Sub-Saharan African context. Thus, knowledge of the readiness of healthcare providers to screen for IPV in the Sub-Saharan African context, as well as of their screening behavior per se remains elusive. This study sets the foundation to fill this knowledge gap by validating the DVHPSS for use in Nigeria.

Specifically, this study will assess the structural validity of the DVHPSS in terms of its factorial structure and sub-scale reliability.

## Methods

**Study settings, design and participants**

This study was carried out at the Amino Kano Teaching Hospital, in Kano, Nigeria, which is the largest multi-departmental federal health institution in Kano state. The staff and patients have a multi-ethnic background and speak English, the official language in the country. In general, the staff members at this hospital have not undergone any specific training in screening for IPV. All Health Care Providers having regular contact with patients (i.e. n = 430) were informed of the study by department heads and invited to participate. Self-administered questionnaires were sent to the eligible participants of which two hundred and seventy four (response rate of 64%) returned the completed questionnaire. Voluntary participation was emphasized and informed consent given. The participants included Psychiatrists, Obstetricians and Gynecologists, Pediatricians, Physicians, Laboratory Scientists, Opticians, Nurses and Midwives. Only those laboratory employees who sometimes meet patients were included. The professions with less than 5 participants (i.e. opticians and laboratory Assistants) were grouped under ‘’others’’. (Table 1). shows some demographic and occupational characteristics of the participants.

**Table:** Table 1: **Characteristics of Participants**

	N	%
Profession		
Doctor	156	58
Nurse	61	22.7
Midwife	12	4.4
Social worker	29	10.8
Others	11	4.1
Gender		
Male	147	56.5
Female	113	43.5
Age (years)		
21-30	116	45.1
31-40	105	40.9
41-60	36	14.0
Marital status		
Married	145	55.1
Single	108	41.1
Divorced	6	2.3
Separated	4	1.5
Religion		
Muslim	114	42.7
Catholic	47	17.6
Protestant	76	28.5
Others	30	11.2
Ethnicity		
Hausa	84	31.5
Ibo	70	26.2
Yoruba	28	10.5
Others	85	31.8
Department		
Medicine	41	15
Surgery	41	15
Pediatric	44	16.1
Obstetrics/Gynecology	36	13.2
General practice	81	29.7
Others	30	11

N = absolute number

% = percentage of total within the group

**Ethical consideration**

This study received ethical approval from the Nigerian Institute of Medical Research, Lagos, Nigeria and the authorities of Aminu Kano Teaching Hospital, Kano. The aims and relevance of the study were further emphasized in a separate document accompanying the questionnaires. Questionnaires were delivered to all the clinical and laboratory departments within the hospital. Only laboratory employees who sometimes meet patients were included.

Voluntary participation was emphasized, privacy guaranteed and informed consent given. Participants dropped off the filled questionnaires at a special collection point centrally located at the hospital.

**Instrument measures**

The Domestic Violence Health Care Provider Survey Scale measures healthcare providers’ readiness to screen for IPV as well as actual screening activity.^15^ The instrument has been previously validated with promising results in developed countries. This paper attempts to assess its structural validity in the Nigerian context. The questionnaire consists of the following 5 subscales:

The perceived self-efficacy subscale (4 items) which assesses providers’ own perceived efficacy in inquiring about IPV(details in table 2). 

The system support sub-scale (4 items) which assesses healthcare providers’ access to support networks for referral/management of IPV victims (details in table 2).

The professional roles resistant/fear of offending clients sub-scale (6 items) which assesses providers’ opinions on whether inquiries about IPV may conflict with ethical issues governing their communication with clients (details in table 2). 

The blame victim sub-scale (7 items) which assesses providers’ attitudes towards victims(details in table 2).

The victim/provider safety sub-scale (10 items) which assesses provider perception on whether inquiring about IPV from batteres would further jeopardize victim/care provider safety(details in table 2).

All items require the respondent to take a position on specific statements. The response alternatives to each statement range from 1 (strongly disagree) to 5 (strongly agree).

Some of the items (statements) are phrased in a way such that their scores need to be reversed to match with other items in the same scale before any further analysis can be done.

**Statistical analysis**

Exploratory factor analysis using principal component method was performed to test underlying factors and their stability as expressed in the factor loadings. Varimax rotation was applied to limit the number of high loadings under the same factor. This would enhance clearer identification of items emerging under each subscale. Criteria for the number of resulting significant factors was based on Kaiser Criterion and confirmed with screen plots.^15-17^ Items with factor loading of at least 0.30 were considered significant; this is based on criteria for significant correlation.^18^The contribution of each factor in explaining the total variation in the item pool was reported. Significant factors (i.e. those having a highest loading of over 0.30) were tested for internal consistency using Cronbach’s Alpha. Each item was then scrutinized further to assess whether the removal of that item would improve the alpha coefficient. If removal of an item implied improved alpha, that item would be removed and the reliability test re-run without that item. The process would continue until a point of saturation was reached (i.e. removal of additional items would not improve alpha). Alpha coefficients of at least 0.70 were considered significant, a threshold adequate for research purposes.^19,20^The resulting items/scales following the reliability test were then re-examined in a new factor analysis. If any of the highest item loadings was less than 0.30, the process described above (i.e. series of factor analyses and reliability test) would continue until all remaining items loaded at least 0.30, the a priori set threshold. Where double loadings were evident, the item was assigned to the factor under which it loaded highest. Bivariate correlations were run to investigate the factor distinctiveness of the final factor solution.^19,20^

Prior to the above analyses, certain procedures were carried out to clean data. First, only participants who had responded to all items were included in the analyses above as failure to do so may introduce erroneous estimates. Second, items were checked for normality using the skewness statistic and its confidence interval. Skewness statistic of magnitude zero is an indication of perfect symmetry. All analyses were run with SPSS version 16.

## Results

**Data cleansing**

Only participants who had responded to all items of the original scales were assessed in the factor analyses and reliability tests. This resulted in a total of 162 participants (59% of total respondents to the questionnaire). The results of the normality test revealed that the confidence intervals for the skewness statistic for each of the 35 items included, or was close to, zero, suggesting minor or no violation of normality assumptions.

**Initial factorial structure with all items**

When all items of the original instrument were included in the factor analysis, 6 factors emerged based on the Kaiser Criterion (Eigen values > 1) (table 2)..The victim/provider safety subscale of the original instrument emerged as two separate subscales in our data, reflecting victim safety and provider safety respectively. The majority of the items of the original DVHPSS exhibited significant factor loading according to the a priori decided threshold of 0.30 (table 2)..

**Table:** Table 2: Rotated factor loadings for Domestic violence Healthcare Providers survey scales restricted to 6 factors

Components	1	2	3	4	5	6
Professional Role Resistance/Fear of offending the Patients						
I am afraid of offending patients if I ask about their abusive behavior	0.62	-0.04	0.08	0.02	0.08	-0.04
I am afraid of offending the patient if I ask about DV	0.69	0.08	0.02	0.00	-0.10	-0.24
Asking patients about DV is an invasion of their privacy	0.78	0.08	0.16	-0.12	0.06	-0.07
It is demeaning to patients to question them about abuse	0.72	0.18	0.20	-0.03	0.08	0.13
If I ask non-abused patients about DV, they will get very angry	0.34	0.13	0.11	-0.04	0.01	-0.05
It is not my place to interfere with how a couple chooses to resolve conflicts	0.57	0.04	0.10	-0.10	-0.13	0.07
When challenged, batterers frequently direct their anger toward health care providers	0.49	-0.13	0.34	0.09	0.11	0.22
If patients do not reveal abuse to me, then they feel it is none of my business	0.14	0.17	0.07	-0.08	-0.04	-0.22
Blame Victim						
A victim must be getting something out of the abusive relationship, or else he/she would leave.	0.11	0.68	-0.06	0.15	0.06	0.06
People are only victims if they choose to be.	0.06	0.45	0.18	0.18	-0.10	0.07
When it comes to domestic violence victimization, it usually “takes two to tango.”	0.00	0.66	0.10	0.06	0.17	0.16
I have patients whose personalities cause them to be abused.	0.02	0.59	0.26	0.02	-0.11	0.15
Women who choose to step out of traditional roles are a major cause of DV.	-0.06	0.74	0.14	-0.00	0.03	-0.08
The victim’s passive-dependent personality often leads to abuse.	0.17	0.46	0.14	0.20	-0.04	0.08
The victim has often done something to bring about violence in the relationship	0.17	0.62	0.16	0.13	0.34	-0.18
Victim safety						
I think that investigating the underlying cause of a patient’s injury is not part of medical care	0.21	0.02	0.42	-0.04	-0.15	0.11
I feel it is best to avoid dealing with the batterer out of fear and concern for the victim’s safety	0.20	0.12	0.71	-0.05	0.14	-0.04
There is no way to ask batterers about their behaviors without putting the victims in more danger	0.11	0.18	0.81	-0.03	-0.01	0.01
I am afraid if I talk to the batterer, I will increase risk for the victim	0.10	0.15	0.74	0.00	0.00	-0.05
I have ready access to information detailing management of DV	0.13	0.26	0.46	0.37	0.06	0.23
Perceived Self-efficacy						
There are strategies I can use to encourage batterers to seek help.	-0.09	0.09	-0.21	0.74	0.11	0.26
There are strategies I can use to help victims of DV change their situation.	0.03	0.12	-0.06	0.79	0.28	0.05
I feel confident that I can make appropriate referrals for batterers.	-0.07	0.13	0.14	0.64	0.39	0.01
I feel confident that I can make the appropriate referrals for abused patients.	-0.27	0.06	0.21	0.59	0.12	0.05
There’re ways I can ask batterers about their behavior that will minimize risk to the potential victim	0.19	0.17	0.16	0.49	-0.01	0.42
I don’t have the time to ask about DV in my practice	0.03	0.07	-0.00	-0.08	-0.12	-0.15
System support						
I have ready access to medical social workers or community advocates to assist in the management of DV.	0.12	0.11	0.17	0.15	0.72	0.08
I feel that medical social work personnel can help manage DV patients.	-0.07	0.08	-0.07	0.31	0.50	0.36
I have ready access to mental health services should our patients need referrals.	-0.01	0.06	-0.11	0.16	0.72	0.12
I feel that the mental health services at my clinic or agency can meet the needs to DV victims in cases where they are needed.	-0.04	-0.01	0.08	0.21	0.67	0.22
Provider safety						
I feel there are ways of asking about battering behavior without placing myself at risk	-0.02	-0.07	-0.02	0.08	0.17	0.69
I feel I can effectively discuss issues of battering and abuse with a battering patient	-0.16	0.05	-0.03	0.28	0.15	0.77
I feel I can discuss issues of battering and abuse with a battering patient without further endangering the victim	0.06	0.22	0.03	0.01	0.20	0.70
I am reluctant to ask batterers about their abusive behavior out of concern for my personal safety.	0.33	-0.02	0.33	-0.00	0.23	-0.13
There is not enough security at my work place to safely permit discussion of DV with batterers	0.06	0.12	0.26	0.04	0.02	0.09
Eigenvalues	5.98	5.06	2.22	1.88	1.67	1.51
% of Variance	17.09	14.47	6.35	5.37	4.77	4.31

Note: Factors loading over 0.30 appear in bold. The extraction method used was Principal component Analysis and rotation method: Varimax rotation with Kaiser Normalization (eigenvalues >1).

Factor 1 emerged as professional role resistant/fear of offending the patients subscale with all but two items of the original scale loading significantly under this factor. Moreover, one item that in the original instrument belonged to the provider safety scale (i.e. “when challenged, batterers frequently direct their anger towards healthcare providers) now loaded significantly under factor 1. This factor included a total of 7 items and explained 17% of the variation in the total item pool.

Factor 2 emerged as Blame victim subscale with all 7 items of the original scale loading significantly under the same. The factor explained about 14.5% of the variation in the total item pool.

Factor 3 reflected a victim safety subscale component, though two additional items from the originally perceived self-efficacy and professionally resistant subscales respectively (i.e. “I have ready access to information detailing management of domestic violence” and “I think that investigating the underlying cause of a patient’s injury is not part of medical care”) now loaded significantly under this factor. This factor included a total of 5 items and explained 6.5% of the variation in the total item pool.

Factor 4 emerged as a perceived self-efficacy subscale with all but two of the items in the original subscale loading significantly under this factor. This factor included a total of 5 items and explained about 5.4% of the variation in the total item pool.

Factor 5 emerged as system support subscale with all 4 items of the original scale loading significantly under this factor. The factor explained about 4.8% of the variation in the total item pool.

Factor 6 reflected a Provider safety subscale with three of the original six items in the victim/provider safety scale loading significantly under this factor. The factor explained 4.3% of the variation in the total item pool.

**Internal reliability**

Only items with highest loadings of 0.30 and above were subjected to a reliability test. Thus, 4 items of the total 35 were dropped (i.e. “I don’t have time to ask about Domestic Violence in my practice”, “If patients do not reveal abuse to me, then they feel it is none of my business”, “There is not enough security at my work place to safely permit discussion of domestic violence with batterers” and “I am reluctant to ask batterers about their abusive behavior out of concern for my own safety”).

As indicated in (table 3)., the internal reliability of the 7 items of the professional role resistant/fear of offending the patients subscale (meeting above criteria) was 0.80. Removal of additional items from this scale would only reduce the internal reliability as expressed in the column “Cronbach’s alpha if item removed” of table 3. In addition, the internal reliability of the 7 items of the Blame victim subscale (meeting above criteria) was 0.77. Removal of additional items from this scale would only reduce the internal reliability. The internal reliability of the 5 items of the new victim safety subscale was 0.73. However, removal of two of these items (i.e. “I have ready access to information detailing management of domestic violence” and “I think that investigating the underlying cause of a patient’s injury is not part of medical care”) was bound to improve alpha. Thus, when these items were removed alpha increased to 0.78. The internal reliability of the 5 items of the perceived self-efficacy subscale was 0.77. Removal of additional items from this scale would only reduce the internal reliability. Likewise, the internal reliability of the 4 items of the system support subscale was 0.73. Removal of additional items from this scale would only reduce the internal reliability. Finally, the internal reliability of the 3 items of the provider safety subscale was 0.72. Removal of additional items from this scale would only reduce the internal reliability.

**Table:** Table 3: **Internal Reliability scores for items of Domestic violence Healthcare Providers survey scales**

Scales items	No of scale items	Cronbach’s Alpha	Cronbach’s Alpha*
Factor 1: Professional Role Resistance/Fear of offending the Patients scale	7	0.80	
Asking patients about DV is an invasion of their privacy			0.78
It is demeaning to patients to question them about abuse			0.76
If I ask non-abused patients about DV, they will get very angry			0.77
I am afraid of offending the patient if I ask about DV			0.79
I am afraid of offending patients if I ask about their abusive behavior			0.77
It is not my place to interfere with how a couple chooses to resolve conflicts			0.76
When challenged, batterers frequently direct their anger toward health care providers			0.80
Factor 2: Blame Victim scale	7	0.77	
A victim must be getting something out of the abusive relationship, or else he/she would leave.			0.74
People are only victims if they choose to be.			0.75
When it comes to domestic violence victimization, it usually “takes two to tango.”			0.73
I have patients whose personalities cause them to be abused.			0.75
Women who choose to step out of traditional roles are a major cause of DV.			0.74
The victim’s passive-dependent personality often leads to abuse.			0.76
The victim has often done something to bring about violence in the relationship			0.75
Factor 3: Victim safety scale	5	0.73	
There is no way to ask batterers about their behavior without putting the victims in more danger			0.58
I am afraid if I talk to the batterer, I will increase risk for the victim			0.72
I feel it is best to avoid dealing with the batterer out of fear and concern for the victim’s safety			0.78
I have ready access to information detailing management of DV			0.78
I think that investigating the underlying cause of a patient’s injury is not part of medical care			0.78
Factor 4: Perceived Self-efficacy scale	5	0.77	
There are strategies I can use to encourage batterers to seek help.			0.72
There are strategies I can use to help victims of DV change their situation.			0.68
I feel confident that I can make appropriate referrals for batterers.			0.72
I feel confident that I can make the appropriate referrals for abused patients.			0.75
There’re ways I can ask batterers about their behavior that will minimize risk to the potential victim			0.76
Factor 5: System support scale	4	0.73	
I have ready access to medical social workers or community advocates to assist in the management of DV.			0.68
I feel that medical social work personnel can help manage DV patients.			0.69
I have ready access to mental health services should our patients need referrals.			0.65
I feel that the mental health services at my clinic or agency can meet the needs to DV victims in cases where they are needed.			0.67
Factor 6: Providers safety scale	3	0.72	
I feel I can effectively discuss issues of battering and abuse with a battering patient.			0.54
I feel there are ways of asking about battering behavior without placing myself at risk			0.64
I feel I can discuss issues of battering and abuse with a battering patient without further endangering the victim			0.71

* If item is removed

DV= Domestic Violence

In total therefore, the reliability test dropped an additional two items.

**Final factorial structure**

The items emerging from the reliability test (i.e. 29 items) were now subjected to a new factor analysis. As indicated in table 4, six factors emerged based on Kaiser Criterion (Eigen values > 1) and all items now loaded significantly (i.e. highest factor loadings of at least 0.30) under respective factor. The explanatory power of each factor in explaining the total variation in the item pool is also reported intable 4.

**Table:** Table 4. **Emerging factor loadings for Domestic violence Healthcare Providers survey scales**

Components	1	2	3	4	5	6
Professional Role Resistance/Fear of offending the Patients						
I am afraid of offending patients if I ask about their abusive behavior	0.63					
I am afraid of offending the patient if I ask about DV	0.70					
Asking patients about DV is an invasion of their privacy	0.79					
It is demeaning to patients to question them about abuse	0.73					
If I ask non-abused patients about DV, they will get very angry	0.41					
It is not my place to interfere with how a couple chooses to resolve conflicts	0.61					
When challenged, batterers frequently direct their anger toward health care providers	0.45					
Blame Victim						
A victim must be getting something out of the abusive relationship, or else he/she would leave.		0.70				
People are only victims if they choose to be.		0.64				
When it comes to domestic violence victimization, it usually “takes two to tango.”		0.74				
I have patients whose personalities cause them to be abused.		0.62				
Women who choose to step out of traditional roles are a major cause of DV.		0.53				
The victim’s passive-dependent personality often leads to abuse.		0.50				
The victim has often done something to bring about violence in the relationship		0.41				
System support						
I have ready access to medical social workers or community advocates to assist in the management of DV			0.75			
I feel that medical social work personnel can help manage DV patients.			0.56			
I have ready access to mental health services should our patients need referrals.			0.64			
I feel that the mental health services at my clinic or agency can meet the needs to DV victims in cases where they are needed.			0.67			
Perceived Self-efficacy						
There are strategies I can use to encourage batterers to seek help.				0.69		
There are strategies I can use to help victims of DV change their situation.				0.76		
I feel confident that I can make appropriate referrals for batterers.				0.67		
I feel confident that I can make the appropriate referrals for abused patients.				0.59		
There’re ways I can ask batterers about their behavior that will minimize risk to the potential victim				0.43		
Victim safety						
I feel it is best to avoid dealing with the batterer out of fear and concern for the victim’s safety					0.72	
There is no way to ask batterers about their behavior without putting the victims in more danger					0.82	
I am afraid if I talk to the batterer, I will increase risk for the victim					0.76	
Provider safety						
I feel there are ways of asking about battering behavior without placing myself at risk						0.68
I feel I can effectively discuss issues of battering and abuse with a battering patient						0.77
I feel I can discuss issues of battering and abuse with a battering patient without further endangering the victim						0.77
Eigenvalues	5.47	4.61	2.20	1.58	1.52	1.29
% of Variance	18.24	15.37	7.32	5.29	5.06	4.31
						

**Inter-factor correlation**

As indicated by the bivariate correlations in table 5, significant correlations ranging in magnitude between 0.17-0.53 were found between most factors.

**Table:** Table 5. **Bivariate Pearson Correlations of Domestic Violence healthcare Providers Survey Scales**

	Professional Role	Blame Victim	System Support	Victim Safety	Self- efficacy	Provider Safety
Professional Role	1.000					
Blame Victim	0.257**					
System Support	0.006	0.198*				
Victim Safety	0.406**	0.382**	0.060			
Self-efficacy	-0.081	0.320**	0.528**	0.075		
Provider Safety	0.049	0.171*	0.424**	0.021	0.431**	1.000

**Correlation is significant at 0.01 level (2-tailed)

* Correlation is significant at 0.05 level (2-tailed)

## Discussion

The factorial structure and internal reliability of the Domestic Violence Healthcare Provider Survey Scales (DVHPSS) in a sample of Nigerian healthcare providers was tested and the instrument to a small extent refined following a priori defined criteria for inclusion. The analysis was thus designed so as to maintain only items an factors that met these criteria. The results in many regards mirrored that of the original instrument. The factors “blame the victim” and “system support” in the original questionnaire was confirmed in our data without any exceptions (i.e. all items in the original subscales met inclusion criteria in our data). Though the other factors did not differ substantially from the original scales, some important observations warranting acknowledgement were made.

First, the victim/provider subscale that formed a distinct sub-scale in the original instrument was split up in our data into two separate scales i.e. victim safety and provider safety subscales. This suggests that healthcare providers in the setting studied recognize victim and provider safety as two distinct aspects of screening for IPV unlike their peers in the developed countries.^15^ In addition, some aspects related to victim and provider safety (i.e. “There is not enough security at my work place to safely permit discussion of domestic violence with batterers” and “I am reluctant to ask batterers about their abusive behaviour out of concern for my own safety”) were dropped out subsequently in the analysis as they did not meet the a priori conditions set. Noteworthy here is that these factors were concerned with how to deal with the perpetrators and not the victims. A plausible explanation as to why these issues loaded low could be that respondents may 	not have identified with these questions. Indeed, it may be difficult to identify batterers among patients, precluding an assessment of batterer-related security risk. Moreover, in the Nigerian context men rarely accompany their partners/children to hospital making access to the potential batterers difficult, particularly in reproductive health and pediatric units.

Second, the perceived self-efficacy and professional role resistance scales lost three items that had to do with lack of time for IPV inquiry, access to information to manage IPV, and challenging whether IPV is part of the medical practice. The reason why these items loaded low is difficult to explain and deserves further scrutiny in future work. However, a plausible explanation, at least for the later two, is that IPV inquiry is not yet an integral part of healthcare practice at this facility. An understanding of what access to information on how to manage IPV implied or the role of IPV inquiry in medical practice might have varied, leading to random response to these items. Introducing protocols on IPV inquiry at this facility in the future thus could help align providers understanding of such issues.

To assess factor distinctiveness, bivariate correlations were established between the emerging factors. These correlations, though statistically significant, ranged from low to moderate, indicating that though inter-related, the factors represent rather distinct aspects of provider readiness to screen.

The current study attempted to assess the usefulness of an already existing instrument (developed in the western world) in the Nigerian context and found the instrument applicable based on its structural validity. However, the item pool used in this study may not be exhaustive of challenges to screening for IPV in the Nigerian context. Qualitative studies could reveal additional challenges to screening specific to the Nigerian context. Indeed dissimilar cultural values could imply unique sets of challenges to screening in each culture. For example, recent studies on violence indicate that in many Sub-Saharan countries including Nigeria, wife beating remains an acceptable societal norm.^21^How such norms may pose specific challenges to screening both from a patient and provider perspective remain to be studied. In addition, other aspects of the validity of the instrument (e.g. concurrent and discriminate validity) may help further validate the usefulness of this instrument in the Nigerian context. These aspects are currently being studied separately.

In conclusion, the factorial stability and internal reliability of the DVHPSS scale in the Nigerian sample has to a large extent been confirmed and can therefore be used to scrutinise readiness to screen for IPV among Nigerian healthcare professionals. However, the Nigerian healthcare providers made an important distinction between victim and provider safety unlike peers in the developed countries.^21^ In addition, batterer-related aspects of screening did not figure as significant factors in this analysis, suggesting that cultural specific factors could play a role. Finally, issues pertaining as to whether IPV should be considered a part of medical practice or access to information on IPV management did not specifically load under any factor, suggesting that the question may have been variedly understood. Clarification of what screening implies (e.g. using protocols) may be useful to align healthcare providers’ understanding of these items. Further investigation of the other aspects of validity is warranted to understand occupational, demographic and cultural aspects that could impede or promote screening activity in this context.
